# Benign Endocervical Polyp with Heterologous Elements in a 42-year-old Female: Report of a Case

**DOI:** 10.7759/cureus.6045

**Published:** 2019-10-31

**Authors:** Miglena K Komforti, Kathleen Whitney, Sun Chung

**Affiliations:** 1 Pathology, Cleveland Clinic Ohio, Cleveland, USA; 2 Pathology, Montefiore Medical Center, Bronx, USA

**Keywords:** benign, endocervical polyp, choristoma, heterologous, heterotopic, adipose, fat

## Abstract

A 42-year-old female with unremarkable medical history presented for a routine cervical screening upon which an endocervical polyp was identified and submitted entirely for histopathologic evaluation. Microscopic examination showed multiple well-circumscribed nodular fragments of polyp with superficial erosion and focal acute inflammation. Benign endocervical glands were seen within a fibrotic stroma with a prominent smooth muscle component. Intermixed mature adipose tissue and large thick-walled vessels were also identified. Stromal and epithelial atypia were absent; similarly, stromal cellularity, mitoses, and condensation were not identified. Additional deeper levels did not reveal other heterologous elements. The diagnosis of a benign choristomatous endocervical polyp was rendered. As anticipated, the patient recovered completely.

A review of the English literature demonstrates rare, namely two other reports of hamartomatous tissue in an endocervical polyp. To the best of our knowledge, we report the third such case.

## Introduction

Endocervical polyps (ECPs) are routine and common specimens in gynecologic pathology. Microscopically, these benign proliferations show a fibrovascular stalk and endocervical glands, and are on occasion accompanied by squamous metaplasia, chronic inflammation, or ulceration if irritated. Herein, we illustrate an unusual histopathologic presentation of an ECP in a 42-year-old female who presented for a routine well-woman visit.

The patient has an otherwise unremarkable medical history. On physical examination and cervical cytology screenings, she was found to have no abnormalities. However, due to the presence of a high-risk human papilloma virus (HPV) on HPV co-testing, she underwent colposcopy during which a cervical polyp was visualized. Polypectomy was performed, and the specimen was sent to pathology.

## Case presentation

Grossly, a 2.5 x 1.9 x 1.0 cm nodular rubbery polyp was received, which was then trisected and submitted entirely for microscopic examination in three blocks. Four micron-thick sections of formalin-fixed paraffin-embedded tissue were prepared with routine hematoxylin and eosin staining protocol.

**Figure 1:**
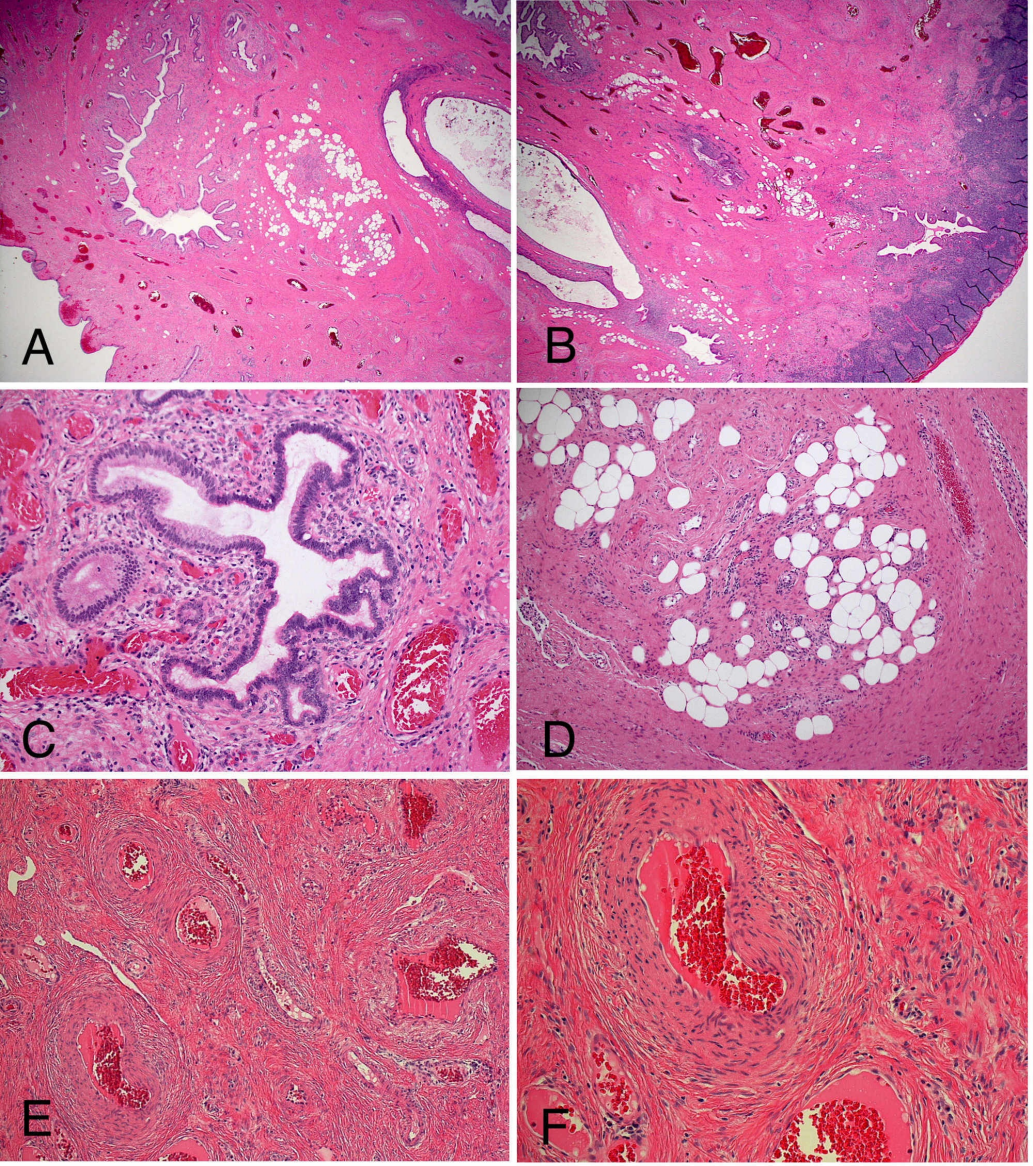
Endocervical polyp with abnormal thick-walled vessels, and heterologous adipose tissue and myofibroblastic stroma A, B. Low power view of two sides of one large section of endocervical polyp, demonstrating focal superficial erosion accompanied by a prominent lymphoplasmacytic aggregate beneath the overlying epithelium, a mixture of smaller and larger dilated endocervical glands, congested dilated and thick-walled vessels, adipose tissue, and myofibroblastic stroma (hematoxylin and eosin [H/E]; original magnification, 2x). C. A closer look at the cytomorphologically bland endocervical glands, surrounding stroma, and dilated vascular channels (H/E; original magnification, 20x). D. Unremarkable mature adipocytes interspersed within myofibroblastic stroma (H/E; original magnification, 10x). E, F. Prominent and abnormally thick-walled vessels within the endocervical polyp stroma (H/E; original magnification, 10x and 20x).

Microscopic examination demonstrated fragments of nodular, well-circumscribed cervical tissue with smooth outer surface. The superficial endocervical epithelium was morphologically benign with focal erosion accompanied by a patchy superficial mixed inflammatory infiltrate (Figure 1A, 1B). The deeper stroma of the polyp also showed benign endocervical glands in a background of smooth muscle, fibrous stroma, and scattered foci of cytologically bland and mature adipocytes (Figure 1C, 1D). Thick-walled vessels were prominent (Figure 1E, 1F).

No stromal atypia or stromal mitoses were identified. Additional deeper sections were examined on each of the three blocks but other heterologous stromal elements were not identified. The diagnosis of benign ECP with hamartomatous elements was rendered.

## Discussion

ECP are thought to represent a benign hyperplastic phenomenon of both the epithelium and underlying cervical stroma. ECP are an anticipated routine specimen in the daily selection of gynecologic pathology, and microscopically often show a loose, edematous stroma, large dilated or small thick-walled vasculature. A fair number of unremarkable endocervical glands are also normally identified. Depending on the extent of irritation, particularly in larger lesions protruding through the cervical os, a certain amount of acute and chronic inflammation, erosion, or even granulation tissue usually localized to the surface is anticipated.

ECP with benign heterologous elements are extraordinarily rare. After a review of the English literature, two case reports, both describing ECP with mature adipose tissue and abnormal thick-walled vessels, were identified [1,2]. Ilhan and colleagues are first to report a 33-year-old female in which a hamartomatous ECP demonstrated mature cartilage and adipose tissue; in addition, focal pseudodecidual change and mesonephric glands were seen [1]. Given the presence of mesonephric glands, this polyp could have represented a benign mixed Müllerian tumor, such as lipoadenofibroma - another rare entity. More recently, Pecorella et al. described a second similar polypoid lesion in a 24-year-old female [2]. That ECP showed benign overlying squamous epithelium, abnormal fibrous stroma with mature adipose tissue but no endocervical glands at all, which are histopathologic findings rather consistent with an angiolipomatous polyp [2]. Common histopathologic findings present in both reports by Ilhan and Pecorella [1,2], as well as the ECP we report herein, are mature adipocytes and thick-walled vessels. Furthermore, Ilhan et al. noted the absence of an internal elastic lamina in the aforementioned vessels [1]. Lastly, a third report mentions cartilage and osseous metaplasia within a large ECP but no mention of the vasculature [3]. In none of the four reports, to include ours, stromal condensation, stromal atypia, or mitoses were seen [1-3].

As it is such an extraordinary event, the presence of heterotopia in the cervix is perplexing to most practicing pathologists. As non-pathological or mass-forming heterotopic tissue, fat and cartilage have previously been described at this site and despite encountering major challenges in accurately identifying adipocytes in the cervix, some studies claim fat can be seen in 15% of cone/loop electrosurgical excision procedures or hysterectomies [4]. Encapsulated mass-forming adipose tissue, a defining feature of lipomas, has extraordinarily been reported in the cervix [5]. Fat has also been reported as part of the rare lipoleiomyoma of the uterus [5]. Other rare heterotopic tissues, namely sebocytes, melanocytes, and neuroglia, have been defined previously as well [6-9].

The origin of heterotopia is obscure, some speculating it to arise either from metaplasia of Wolffian duct remnants or from chondroid differentiation of the uterine stroma [6]. Additional alternative theories include metaplasia from endocervical stromal stem cells, direct transformation of smooth muscle cells via intracellular accumulation of lipids, or even fetal origin. However, the presence of atypical large thick-walled vessels without an elastic lamina, as described by Ilhan et al., makes the latter theories less likely, and is rather in support of hamartomatous origin [1].

The identification of choristomatous ECP with the unusual and rare finding of adipocytes, cartilage, and abnormal large vessels within the stroma can be worrisome to the practicing pathologist. When obvious endocervical glands are not identified within the polyp, as illustrated by Pecorella, or mesonephric glands are noted, as per Ilhan [1,2], the diagnosis may become even more challenging. Most importantly, the identification of hamartomatous elements should prompt the pathologist to exclude other mimickers, particularly malignant neoplasms. The differential diagnosis includes adenosarcoma represented by benign glands with intraglandular projections ad rigid cysts, and malignant stroma in the form of stromal condensation, stromal mitoses, and atypia. While the uterine corpus is the most common location of adenosarcoma, it can also arise in the cervix where it typically forms a polypoid mass with rather solid cut surface. Heterologous elements, most commonly rhabdomyosarcoma but also immature cartilage or smooth muscle metaplasia, may be present. Carcinosarcoma, in which both the glandular and stromal components are frankly malignant, should also be excluded as this aggressive tumor may also arise in the uterine cervix. Heterologous components commonly represented in this tumor include muscle, cartilage, and fat. In younger patients, such as the one we report herein, the diagnosis of carcinosarcoma is less likely. When the epithelial component is benign, as in this case, close attention should be paid to the stromal component to rule out adenosarcoma. Lastly, a mixed or mesenchymal lesion should also be considered. Therefore, regardless of their size and per general practice guidelines, ECP, similar to endometrial polyps, should always be submitted entirely for histopathologic examination.

## Conclusions

We report the case of a choristomatous ECP in a 42-year-old healthy female, which to the best of our knowledge, is the third such case in the English literature. The practicing pathologist and gynecologist should be aware that the presence of benign heterologous elements in an ECP albeit curious and quite rare is indeed possible. Comfort in a benign diagnosis can be found after complete histopathologic examination of the polyp, as is the protocol recommended for endometrial polyps, and most importantly after more common malignant mimickers such as carcinosarcoma and adenosarcoma are excluded.
